# Ant Droplet Dynamics Evolve via Individual Decision-Making

**DOI:** 10.1038/s41598-017-13775-5

**Published:** 2017-11-01

**Authors:** Tomoko Sakiyama

**Affiliations:** Graduate School of Natural Science and Technology, Okayama University, Okayama, 700-8530 Japan

## Abstract

The droplets of a set of ants were studied while they constructed a bridge. A droplet is a group of ants derived from a larger group. Several experimental studies have revealed the droplet dynamics of ants that resemble the self-organising characteristics that are displayed in their physico-chemical systems. However, little is known regarding how these typical behaviours emerge from individual decision-making. In this study, I developed an agent-based model where artificial ants aggregated, thereby resulting in chain and droplet growth. In my proposed model, the agents tuned their weight thresholds according to the local pattern stability and propagation of negative information. As a result, it was revealed that the droplet dynamics of my proposed model partly matched the time series of droplets of real ants, as demonstrated in previous experimental studies that included the fluctuation function and interdrop increments that followed a scale-free distribution.

## Introduction

Ants communicate directly or indirectly with their nest mates. They use chemical elements (pheromones), body interactions, tandem running and other means^1–3^. The mass behaviour of a group, namely, collective behaviour seems to emerge from simple mechanisms at the agent level. In that sense, collective behaviour of ants can be emergent behaviour, i.e., growth or evolution of more complex forms through simple rules. Collective behaviours of ants appear to display self-organising properties similar to non-living systems^4^. Self-organization is defined as a process by which systems that are composed of many parts spontaneously acquire their structure. For example, mass recruitments of ant foragers display non-linear responses at bridge bifurcations, one of the most famous self-organised emergent behaviours^5–7^.

Although ant societies demonstrate properties displayed in physico-chemical systems, there is a crucial difference between them. In a previous study, researchers pointed out the ability of each ant individual to manage and process information about environmental and social parameters, to suitably tune its interactions with nest mates^4^. Even though living systems apparently behave like chaotic, non-living systems, the mechanisms of self-organized behaviours are not necessarily the same. To this end, mechanisms of self-organised behaviours of ants resembling those of non-living systems may be revealed through decisions made by ant individuals.

Grouping patterns in ants have also been studied as examples of collective behaviours^8–12^. These patterns also seem to display self-organising properties, similar to non-living systems^8^. In recent studies, however, it was revealed that masses of fire ants show duality, i.e. groups of fire ants are able to behave like a liquid or a solid, dependent on the situation^3,9^. They appear to adjust their links to other nearby ants, based on the surrounding environment. Fire ants are also known to build tower-like structures. The ant tower shows a bell shape, which is different from a pile of dead ants that has a conical shape^13^. This example shows that the individual behaviours of living particles affect the macro properties of the ensemble.

Single drops of a set of ants (droplets), a group pattern, have been observed when an aggregation of ants constructs a bridge^10–12^. However, droplet-building behaviour in ants is unexplored. Little is known how decision-making of individuals contributes to self-organised behaviours resembling those of a non-living system, e.g. a dripping faucet. Therefore, the aim of this study is to develop an agent-based model that illustrates droplet growth of ants. When designing an agent-based simulation, an important question to answer is how to model the decision making processes of the agents in the system even if that system looks like another system at first glance and at the macro-level. In this study, I developed two-dimensional droplet growth models of artificial ants and introduced decision-making by agents when they connect to other agents, resulting in the growth of droplets of agents. In my model, artificial ants coordinate their weight threshold and then disconnect or try to hold their links to droplets of other agents. As a result, I observed phenomena in my model that partly correspond to those found in experiments with ants in respect with time series of droplets^11^.

## Materials and Methods

### Space and agents

Artificial agents that move in a two-dimensional discrete lattice were simulated. Cartesian coordinate system was assumed. Φ agents enter the system per unit time from a rod that is one lattice site. As boundary conditions, agents are not allowed to locate above the rod once they enter the system. In each sub-model, agents update their positions synchronously.

### Sub-models

#### Move down

After entering the system, agents may move in three directions (−*y*, +*x*, −*x*), with the following probabilities, until they encounter an unoccupied lattice in each unit time.

1 $${x}_{{\rm{t}}+1}={x}_{{\rm{t}}},\quad {y}_{{\rm{t}}+1}={y}_{{\rm{t}}}-1,\quad \quad {\rm{with}}\,Pro{b}_{{\rm{y}}-}$$

2 $${x}_{{\rm{t}}+1}={x}_{{\rm{t}}}+1,\quad {y}_{{\rm{t}}+1}={y}_{{\rm{t}}},\quad \quad {\rm{with}}\,Pro{b}_{{\rm{x}}+}$$

3 $${x}_{{\rm{t}}+1}={x}_{{\rm{t}}}-1,\quad {y}_{{\rm{t}}+1}={y}_{{\rm{t}}},\quad \quad {\rm{with}}\,Pro{b}_{{\rm{x}}-}$$

After reaching an unoccupied lattice, agents stay immobile. Only a single immobile agent can occupy one lattice. I defined immobile agents as ‘inactive’ agents relative to agents that move (active agents). Thus, each agent is set as an active agent at the beginning of each trial. Active agents are able to move on inactive agents, resulting in droplet and chain growth. Note that this sub-model is only for active agents.

#### Weight calculation

**Figure 1:**
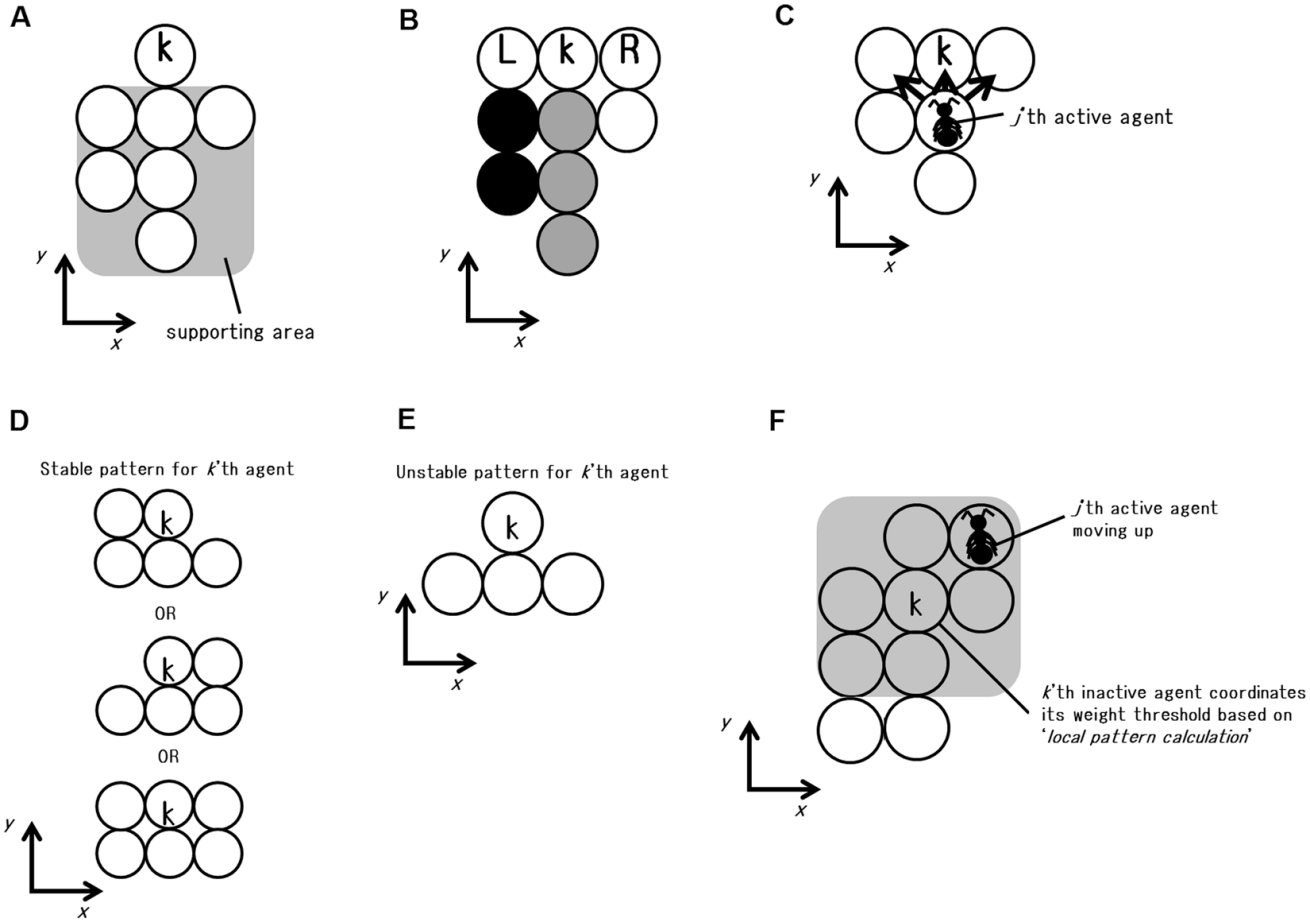
Illustrations of sum-models. Circles represent inactive agents. A and B. ‘*weight calculation’*. (**A**) The supporting area. The grey zone indicates the supporting area for the labelled inactive agent (*k*’th agent). (**B**) A schematic illustration of weight sharing. For the *k*’th agent, the weight of three grey circle agents is equally shared by two neighbours (labelled *R* and *L*) and the weight of two filled circle agents is equally shared by the left-side neighbour. The weight of one unfilled circle agent is equally shared by right-side neighbour. In this example, weight for the *k*’th agent and two neighbours can be 2.5, 2.0 (*L*) and 1.5 (*R*) respectively. (**C**) A schematic illustration of *‘Warning signal’*. An object shaped like an ant represents an active agent (*j*’th agent). If the *k*’th inactive agent signals, then the *j*’th active agent randomly moves in one direction from other three directions illustrated by three arrows. (**D** and **E**) ‘*Local Pattern calculation*’. (**D**) A schematic illustration of stable pattern for the *k*’th inactive agent. (**E**) A schematic illustration of unstable pattern for the *k*’th inactive agent. (**F**) ‘*Threshold coordination*’. If there is at least one active agent (*j*’th agent) that is moving up in the grey zone, then the *k*’th inactive agent coordinates its weight threshold according to the local pattern recognition.

Here, I describe how to compute the weight supported by agents. Inactive agents (immobile agents) support agents under them. The supporting area for each inactive agent is defined in Fig. 1A. Weights for inactive agents are defined by the total number of agents (both inactive and active agents) within the supporting area. Thus, Active agents below inactive agents are also counted as part of the burden borne. If a set of agents defined by one supporting area is connected by a single link, the total weight of a set is assigned to the single agent. Otherwise, several inactive agents share the weight of a set when multiple links support the set. For example, each inactive agent supports one-half or one-third of the weight of a shared supporting area if two or three, respectively, different inactive agents share part of the supporting area of a set (see Fig. 1B). Ants are not necessarily linked to each other in the horizontal direction. Rather, they appear to be linked to each other in the vertical direction. Therefore, I set the supporting area as above.

As shown in the following equations, if an inactive agent is located at the end of an unstable horizontal structure, although rare, that agent is removed from the system (Please see Figure S1). Note that this event is not counted as a droplet.

If any *j*’th inactive agent does not satisfy the following situation,

4 $${\rm{abs}}({{x}^{{\rm{j}}}}_{{\rm{t}}}-{{x}^{{\rm{k}}}}_{{\rm{t}}})\le 1,\quad \quad {{y}^{{\rm{j}}}}_{{\rm{t}}}={{y}^{{\rm{k}}}}_{{\rm{t}}}+1,$$

then the *k*’th inactive agent is removed from the system.

#### Warning signal

Real ants occasionally move up when their bridge becomes unstable. I assume that inactive agents signal when their weights become to be high. If this event happens, active agents near signalling agents are allowed to move up in the following manner:

$${\rm{if}}\,weigh{{t}^{{\rm{k}}}}_{{\rm{t}}}\ge (threshol{{d}^{{\rm{k}}}}_{{\rm{t}}}\,-\,1)\,{\rm{for}}\,k\text{'}{\rm{th}}\,{\rm{inactive}}\,{\rm{agent}},$$

then *j*’th active agent satisfying

5 $${{x}^{{\rm{j}}}}_{{\rm{t}}}={{x}^{{\rm{k}}}}_{{\rm{t}}},\quad \quad {{y}^{{\rm{j}}}}_{{\rm{t}}}={{y}^{{\rm{k}}}}_{{\rm{t}}}-1,\quad j\ne k$$

randomly moves in one direction selected from {(*x* ^j^_t_, *y* ^j^_t_ + 1), (*x* ^j^_t_ + 1, *y* ^j^_t_ + 1), (*x* ^j^_t_ − 1, *y* ^j^_t_ + 1)}

Note that active agents are able to move only to occupied lattices. For example, the *j*’th agent that detects a warning signal is not allowed to randomly choose one lattice from {(*x* ^j^_t_, *y* ^j^_t_ + 1), (*x* ^j^_t_^ + ^1, *y* ^j^_t_ + 1), (*x* ^j^_t_ − 1, *y* ^j^_t_ + 1)} but forced to move only to (*x* ^j^_t_, *y* ^j^_t_ + 1) when (*x* ^j^_t_, *y* ^j^_t_ + 1) is an occupied lattice and others are unoccupied.

#### Fall

When one of the inactive agents supporting a set cannot endure the weight, this set falls and is removed from the system as follows:

6 $${\rm{if}}\,threshol{{d}^{{\rm{k}}}}_{{\rm{t}}}\le weigh{{t}^{{\rm{k}}}}_{{\rm{t}}},$$

then a set supported by *k*’th inactive agent is removed from the system;

$$weigh{t}^{{\rm{k}}}\to 0$$

Note that even though some inactive agents supporting that set can endure the weight, their links to that set also fail and their *weight* values are also reset to zero.

#### Local pattern calculation

I assume inactive agents judge bridge local stability throughout the time they share weight with neighbours. If the following situations are satisfied, inactive agents regard local stability of their bridge as stable or unstable.

if *j*’th inactive agent satisfies

7 $${\rm{abs}}({{x}^{{\rm{j}}}}_{{\rm{t}}}-{{x}^{{\rm{k}}}}_{{\rm{t}}})=1,\quad \quad {{y}^{{\rm{j}}}}_{{\rm{t}}}={{y}^{{\rm{k}}}}_{{\rm{t}}},\quad j\ne k$$

where, ‘abs’ means absolute calculation then *k*’th inactive agent recognises local pattern of their bridge as stable one. Else *k*’th inactive agent recognises local pattern of bridge as unstable one. Note that individuals occasionally misunderstand the stability of a bridge because estimated local patterns calculated by individuals do not necessarily match the actual pattern of a bridge, i.e. the collective output of many individual decisions.

#### Threshold coordination

I assume that inactive agents sometimes coordinate their threshold values according to local pattern recognition. If inactive agents regard the local pattern of their bridge as unstable or stable based on ‘*local pattern calculation*’, they change their weight threshold as follows:

if *j*’th active agent moving up based on ‘*warning signal*’ satisfies following equations,

8 $${\rm{abs}}({{x}^{{\rm{j}}}}_{{\rm{t}}}-{{x}^{{\rm{k}}}}_{{\rm{t}}})\le 1,\quad {\rm{abs}}({{y}^{{\rm{j}}}}_{{\rm{t}}}-{{y}^{{\rm{k}}}}_{{\rm{t}}})\le 1,\quad j\ne k$$

if bridge stability estimated from ‘*local pattern calculation*’ is unstable for *k*’th inactive agent,

9 $${\rm{then}}\quad \quad threshol{{d}^{{\rm{k}}}}_{{\rm{t}}}\to weigh{{t}^{{\rm{k}}}}_{{\rm{t}}}$$

else if bridge stability estimated from ‘*local pattern calculation*’ is stable for *k*’th inactive agent,

10 $${\rm{then}}\quad \quad threshol{{d}^{{\rm{k}}}}_{{\rm{t}}}\to threshol{{d}^{{\rm{k}}}}_{{\rm{t}}}+1$$

To this end, inactive agents who estimate a local pattern as unstable replace their weight threshold with the current weight, if there are active agents trying to move up around them. This modification indicates that active agents moving up around inactive agents encourage the latter to regard the local unstable pattern as a global unstable pattern. After modification, inactive agents tend to disconnect their links to a set of agents and try to maintain the rest of the system as stable.

Otherwise, inactive agents increase their weight threshold when the local pattern is estimated to be stable. Active agents moving up around them inform them of global alert information and encourage them to maintain links to a set of agents.

### Model description

Here, I explain each model description. I developed four different models. One is the threshold-modified model (TM model). The other three are the non-threshold-modified model (NTM model), threshold-modified (stable) model (TM-stable model) and threshold-modified (unstable) model (TM-unstable model). These models served as the control models. Each model can be encoded using the above sub-models.

*Threshold-modified model*

STEP 1: Move down

STEP 2: Weight calculation

STEP 3: Warning signals

STEP 4: Threshold coordination

STEP 5: Local pattern calculation

STEP 6: Fall

STEP 7: Go to STEP1 (time step *t* → *t* + 1)

Note that ‘*Local pattern calculation*’ occurs after ‘*Threshold coordination*’. Therefore, past information (obtained at time step *t −* 1) regarding ‘*Local pattern calculation*’ is used for ‘*Threshold coordination*’ at time step *t*.

*Non-threshold-modified model*

STEP 1: Move down

STEP 2: Weight calculation

STEP 3: Warning signal

STEP 4: Fall

STEP 5: Go to STEP1 (time step *t* → *t* + 1)

*Threshold-modified (stable) model*

In this model, equation (9) is removed from the sub-model ‘*Threshold coordination*’. Therefore, agents coordinate their weight threshold only when bridge stability estimated from ‘*local pattern calculation*’ is stable.

STEP 1: Move down

STEP 2: Weight calculation

STEP 3: Warning signals

STEP 4: Threshold coordination (equation (9) is removed)

STEP 5: Local pattern calculation

STEP 6: Fall

STEP 7: Go to STEP1 (time step *t* → *t* + 1)

*Threshold-modified (unstable) model*

In this model, equation (10) is removed from the sub-model ‘*Threshold coordination*’. Therefore, agents coordinate their weight threshold only when bridge stability estimated from ‘*local pattern calculation*’ is unstable.

STEP 1: Move down

STEP 2: Weight calculation

STEP 3: Warning signals

STEP 4: Threshold coordination (equation (10) is removed)

STEP 5: Local pattern calculation

STEP 6: Fall

STEP 7: Go to STEP1 (time step *t* → *t* + 1)

### Parameters

Parameters are shown in Table 1. Later, I will discuss the influence of changing the parameters.

**Table 1:** Default parameters are shown.

Parameter	Value	Description
*Φ*	1	rate of inflow (agent/unit time)
*Prob*_y-_	0.9	probability of going in the −y direction for active agents
*Prob*_x+_	0.05	probability of going in the +x direction for active agents
*Prob*_x−_	0.05	probability of going in the −x direction for active agents
*threshold*	5	weight threshold for inactive agents
*time length*	1000	time length of one trial
*N of trials*	100	number of trials conducted for measurement

## Results

**Figure 2:**
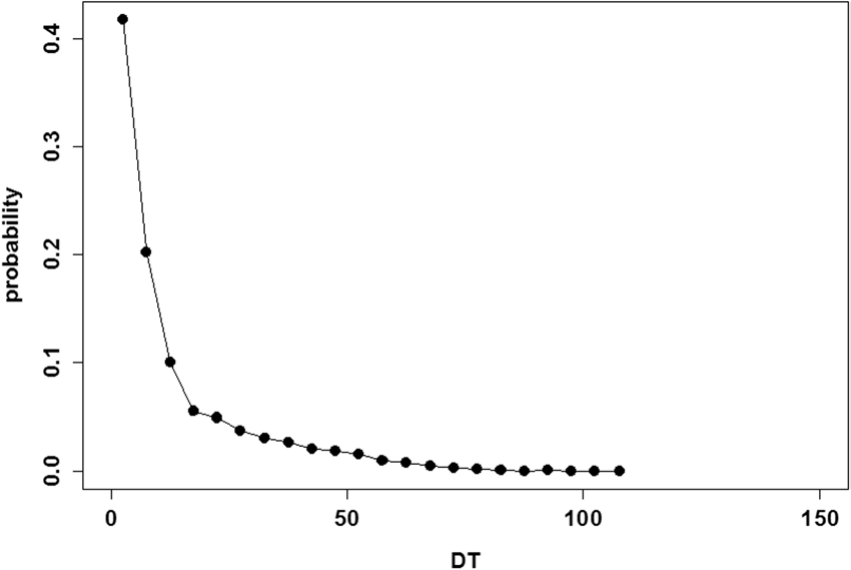
Probability distribution *P* (*DT*) of the interdrop interval *DT*.

Here, I measured time quantities using proposed models. It is well known that droplets of real ants exhibit non-linear dynamics. Let *DT* (*n*) be the time interval between two consecutive droplets ([*n* + 1]’ th and *n*’th droplets). Figure 2 demonstrates the probability distribution *P* (*DT*) of the interdrop interval *DT*. It appears that droplets occur within small intervals, while occasionally, no droplet occurs for a long period. This distribution somewhat matches with the probability distribution *P* (*DT*) that was observed in ant experiments. Several droplets occurred within 10–20 intervals. Averaged droplets size was 12.80 ± 22.46, which was close to that observed in Théraulaz experiments^11^.

**Figure 3:**
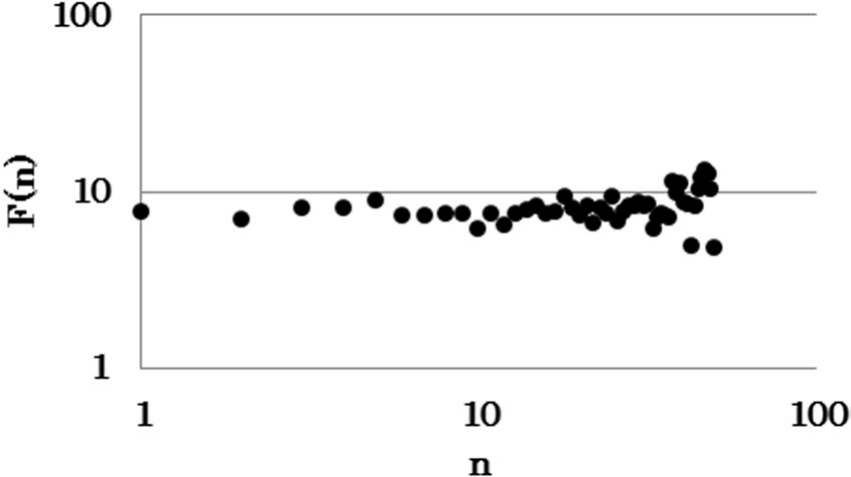
The fluctuation function *F* (*n*) that quantified the magnitude of fluctuations over different scales *n* is shown. Approximately 50 droplets were observed in one trial.

**Figure 4:**
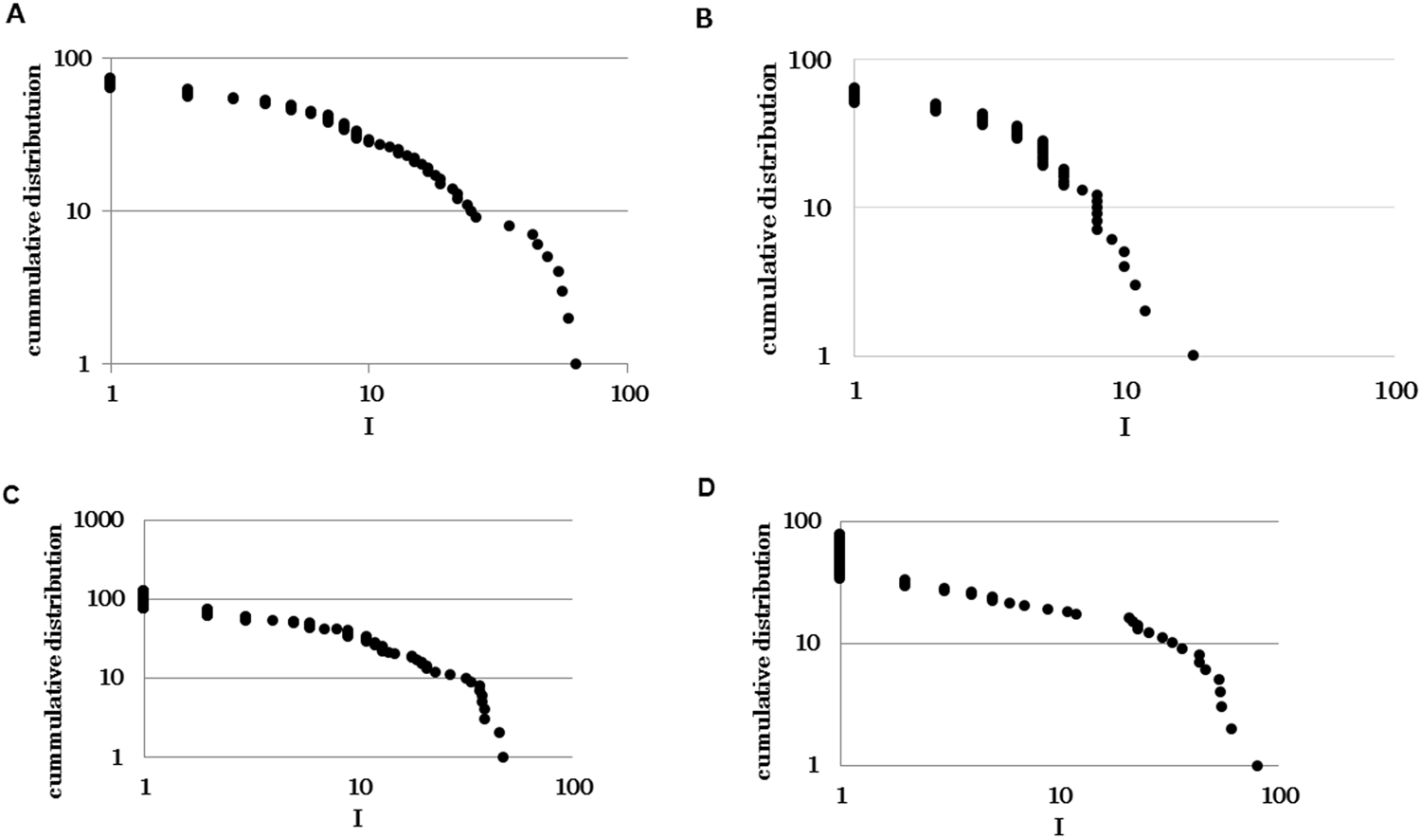
The relationship between increment *I* (*n*) and cumulative distribution of *I* (*n*) are shown. (**A**) The threshold-modified model (threshold = 5 (default). (**B**) The non-threshold-modified model (threshold = 5). (**C**) The threshold-modified model (threshold = 3). (**D**) The threshold-modified model (threshold = 7).

I analysed the fluctuation function that quantified the magnitude of the fluctuations over different scales^13,14^. I observed approximately 50 droplets in each trial. It revealed that *F*(*n*) ∝ *n* ^α,^ with *α* = 0.06 for the TM model (Fig. 3). This result also corresponds with the fluctuation function observed in ant experiments^11^. Moreover, interdrop increments defined by *I* (*n*) = *DT* (*n* + 1) − *DT* (*n*) followed a Lévy distribution in the TM model, which was also observed in previous studies^11,14,15^. Interestingly, the NTM model could not exhibit scale-free properties regarding *I* (*n*) (Fig. 4A, the TM model, number of plotted data = 90, *μ* = 1.63, AIC weights of power-law against exponential-law = 1.00, GOF: *G* = 2.03, d*f* = 3, *P* = 0.57, *NS*, Fig. 4B, the non-threshold-model, number of plotted data = 64, *λ* = 0.29, AIC weights of power-law against exponential-law = 0.00. GOF: *G* = 9.38, d*f* = 5, *P* = 0.095, *NS*, plotted data was collected from five trials for each model.)^16^. The TM-stable and TM-unstable models exhibited considerably different quantities of real ants’ droplets. Both models could not match the probability distribution *P* (*DT*) observed in ant experiments. Droplets appear to occur within large intervals in the TM-stable model. In contrast, droplets appear to occur within very small intervals in the TM-unstable model (see Figures S2 and S3). These results suggest that sub-model ‘*Local pattern calculation*’ and ‘*Threshold coordination*’ are essential.

**Figure 5:**
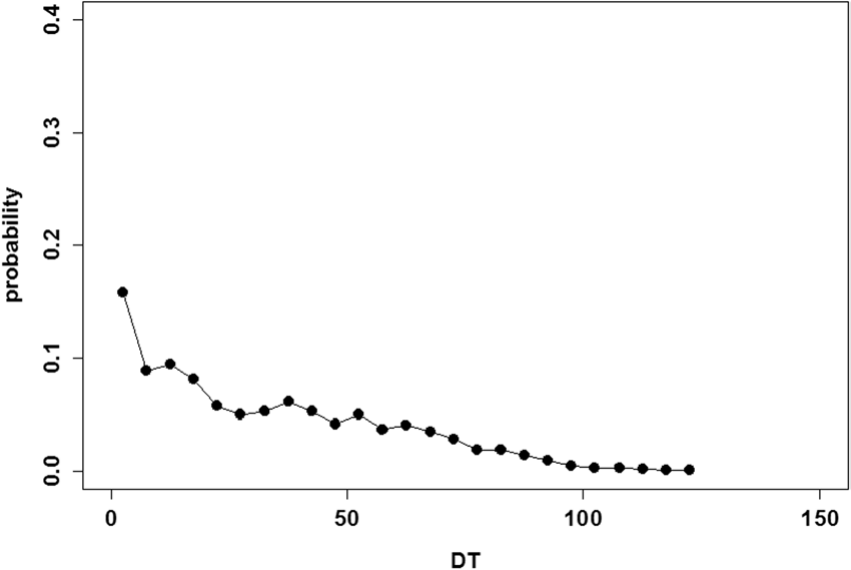
Probability distribution *P* (*DT*) of the interdrop interval *DT* after replacing default parameters with *threshold* = 3, *Prob*_y−_ = 0.50, *Prob*_x+_  = 0.25 and *Prob*_x−_ = 0.25.

I examined how changing parameters influenced the results. Here I fixed the rate of inflow *Φ* for simplicity and appropriately changed other parameters. First, I replaced only a parameter *threshold* = 5 with *threshold* = 3 or 7 and measured *I* (*n*) distribution. According to Fig. 4C and D, Lévy distribution could be maintained even after changing the parameter (Fig. 4C, *threshold* = 3, number of plotted data = 126, *μ* = 1.82, AIC weights of power-law against exponential-law = 1.00, GOF: *G* = 7.85, d*f* = 4, *P* = 0.097, *NS*, Fig. 4D, *threshold* = 7, number of plotted data = 77, *μ* = 1.94, AIC weights of power-law against exponential-law = 1.00, GOF: *G* = 0.79, d*f* = 2, *P* = 0.67, *NS*, plotted data was collected from five trials for each model). Finally, I replaced parameters *threshold* = 5, *Prob*_y−_ = 0.90, *Prob*_x+_ = 0.05 and *Prob*_x−_ = 0.05 with *threshold* = 3, *Prob*_y−_ = 0.50, *Prob*_x+_ = 0.25 and *Prob*_x−_ = 0.25. In this case, even droplet’ intervals differed from those of experimental studies. I found small droplet’ intervals were never achieved in this case, which is different from the quantities of real ants’ droplets (Fig. 5). I found that the averaged maximum aspect ratio for each trial was 12.6:11.6 in the TM model using these parameters. On the other hand, I found the averaged maximum aspect ratio for each trial was 6.2:17.8 in the TM model using default parameters. To this end, it appears to be necessary for active agents to sharply move down in order to produce frequent droplet falls.

Under the natural condition, ants might sometimes enter a droplet from different heights. For instance, ants might enter a droplet from one of several branches when they construct a chain on a tree. To this end, I replaced the entering position from (500, 500)  to (500, 500), (500, 499) and (500, 498). Therefore, the entering position for each agent was randomly selected from the three positions. According to Figure S4, Lévy distribution could be maintained even after the initial configuration was modulated (number of plotted data = 355, *μ* = 1.68, AIC weights of power-law against exponential-law = 1.00, GOF: *G* = 5.30, d*f* = 2, *P* = 0.071, *NS*).

## Discussion

I developed a droplet growth model of artificial ants. In my proposed model, agents sometimes modify their weight threshold (subjective weight) if they perceive indirect warning signals via active agents that are moving up which is coded in a sub-model “*Threshold coordination*”. Here, indirect warning signals indicate that inactive agents sometimes coordinate their weight threshold even though their perceived weights are under the threshold when they were near a signalling agent. Those agents are informed a warning signal (negative information) by active agents who directly receive a warning signal and tend to move up. As a result, I found that the proposed model partly reproduced chaotic phenomenon observed in droplet growth of real ants^10,11^.

In previous studies, models assumed phenomenological results/functions obtained from experimental studies. The probability that a link fails followed the sigmoid function of the total weight supported by that link, which might contribute to scale-free behaviours. However, the probability that a link fails in my proposed model would evolve via ‘*threshold coordination*’, which might be important for achieving typical behaviour. In that sense, my model might describe how to model the decision-making processes of agents in the chain-droplet system of ants using a simple rule. Actually, there is a study regarding macro-behaviour using a simple threshold rule rather than a complicated sigmoidal rule^17^.

Ants can support items that are many times as heavy as their bodies^18^. Considering an individual ant’s load capacity, individual members of ant groups may become less productive when they are surrounded by nest mates, thereby contributing to frequent droplets of sets of ants^19,20^; however, these droplets occasionally do not occur for a while. Moreover, if agents obey the fixed-weight threshold, scale-free properties will never be achieved. To this end, individual ants may endure heavier weights to some extent. In the proposed model (the TM model), these behaviours are implemented via local pattern stability. The contribution of isolated ants appears to decrease compared with that of non-isolated ants in the same size group^18^. Local stable pattern, i.e. the lateral connection between agents, would contribute to strengthen the droplets.

Several studies reported that ants modulate their aggregation abilities^3,8,9,19,21^. For example, fire ants behave not only like a solid but also like a liquid, which appears to be dependent on environmental conditions^3^. Moreover, army ants appear to adjust their bridge in a cost-benefit tradeoff^22^. These results suggest that ants suitably adjust their links to other agents. Also in the TM model, artificial ants adjust the intensity of their weight linkages, resulting in long-term droplet growth on one hand and frequent droplet falls on the other hand.

Bak reported a mechanism for biological learning and adaptation based on extremal dynamics and negative feedback^23^. For any mistake, the strengths of synapses get reduced. In that sense, weight thresholds for activations are modified, which appears to resemble my proposed model because active agents disconnect their links to a set of agents when their weights reach beyond the threshold. In both models, the agents reduce the strengths of synapses/linkages based on their experiences. Nevertheless, agents in our model modify their weight thresholds even without signalling a warning. This modification indicates that surrounding agents, which receive indirect alert information, share the warning signalled by an agent. Therefore, agents in our model sometimes regard an unserious weight as a heavy one when negative information is received.

In my proposed model, inactive agents sometimes disconnect their links to a set of agents even if their perceived weights are under the threshold. Inactive agents occasionally perceive current weight as heavier or lighter. Interpretation of subjective weight might be reconsidered via those propagations, which would contribute to emergent living system behaviours.

## Electronic supplementary material

Supporting Figures

## References

[1] Jackson DE, Ratnieks FLW (2006). Communication in ants. Curr. Biol..

[2] Franks NR, Richardson T (2006). Teaching in tandem-running ants. Nature.

[3] Tennenbaum M, Liu L, Hu D, Fernandez-Nieves A (2016). Mechanics of fire ant aggregations. Nature. Materials..

[4] Detrain C, Deneubourg JL (2006). Self-organised structures in a superorganism: do ants behave like molecules?. Physics of life Reviews.

[5] Deneubourg JL, Aron S, Goss S, Pasteels JM (1990). The self-organizing exploratory pattern of the argentine ant. Journal of Insect Behavior.

[6] Goss S, Aron S, Deneubourg JL, Pasteels JM (1989). Self-organized shortcuts in the argentine ant. Naturwissenschaften.

[7] Deneubourg JL, Goss S, Franks N, Pasteels JM (1989). The blind leading the blind: modeling chemically mediated army ant raid patterns. Journal of Insect Behavior.

[8] Mlot NJ, Tovey CA, Hu DL (2011). Fire ants self-assemble into waterproof rafts to survive floods. Proceedings of the National Academy of Sciences.

[9] Foster PC, Mlot NJ, Lin A, Hu DL (2011). Fire ants actively control spacing and orientation within self-assemblages. Journal of Experimental Biology.

[10] Bonabeau E (1998). Dripping faucet with ants. Phys. Rev. E..

[11] Théraulaz G (2001). Model of droplet dynamics in the argentine ant Linepthema Humile (Mayr). Bulletin of Mathematical Biology.

[12] Lioni A, Sauwens C, Theraulaz G, Deneubourg JL (2001). Chain formation in Oecophylla longinoda. Journal of Insects Behaviour.

[13] Phonekeo S, Mlot N, Monaenkova D, Hu DL, Tovey C (2017). Fire ants perpetually rebuild sinking towers. Royal Society Open Science.

[14] Peng CK (1993). Long-range anticorrelations and non-Gaussian behavior of the heartbeat. Phys. Rev. Lett..

[15] Penna TJP, de Oliveira PMC, Sartorelli JC, Gonçalves WM, Pinto RD (1995). Long-range anticorrelations and non-Gaussian behavior of a leaky faucet. Phys. Rev. E..

[16] Edwards AM (2007). Revisiting Lévy flight search patterns of wandering albatrosses, bumblebees and deer. Nature..

[17] Robinson EJH, Franks NR, Ellis S, Okuda S, Marshall JAR (2011). A Simple Threshold Rule Is Sufficient to Explain Sophisticated Collective Decision-Making. PLOS ONE.

[18] Wojtusiak J, Godzińska EJ, Dejean A (1995). Capture and retrieval of very large prey by workers of the African weaver ant, Oecophylla longinoda (Latreille 1802). Tropical Zoology..

[19] Phonekeo S, Dave T, Kern M, Franklin SV, Hu DL (2016). Ant aggregations self-heal to compensate for Ringlemann. Effect. Soft Matter..

[20] Gelblum A (2015). Ant groups optimally amplify the effect of transiently informed individuals. Nature. Communications..

[21] Hu DL, Phonekeo S, Altshuler E, Brochard-Wyart F (2016). Entangled active matter: From cells to ants. The European Physical Journal Special Topics..

[22] Reid CR (2015). Army ants dynamically adjust living bridges in response to a cost-benefit trade-off. Proceedings of the National Academy of Sciences of the USA.

[23] Bak P, Chialvo DR (2001). Adaptive learning by extremal dynamics and negative feedback. Physical Review E.

