# Supplementary material for: Ant Droplet Dynamics Evolve via Individual Decision-Making
Source: Sci Rep. 2017 Nov 1;7:14877. doi: 10.1038/s41598-017-13775-5 (PMC5666022; doi:10.1038/s41598-017-13775-5)
Supplement: Supplementary file 1 — Supporting Figures [file 41598_2017_13775_MOESM1_ESM.doc]

**Ant Droplet Dynamics Evolve via Individual Decision-Making**

**Tomoko Sakiyama^1^**

**Tomoko Sakiyama^1^, Graduate School of Natural Science and Technology, Okayama University, Okayama, 700-8530, Japan**

**Correspondence:**

**Tomoko Sakiyama,**

**Graduate School of Natural Science and Technology, Okayama University, Okayama, 700-8530, Japan**

[tmk.sakiyama@gmail.com](mailto:tmk.sakiyama@gmail.com)


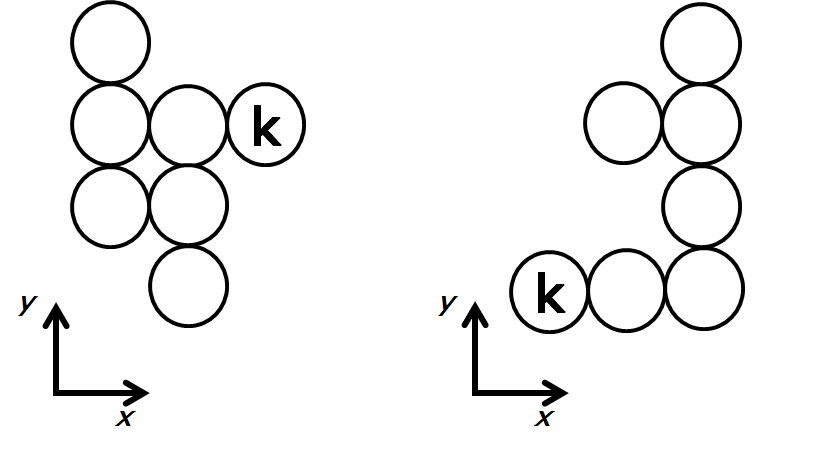


Figure S1. Example configurations of *k*’th inactive agent that is removed from the system. Circles represent inactive agents.


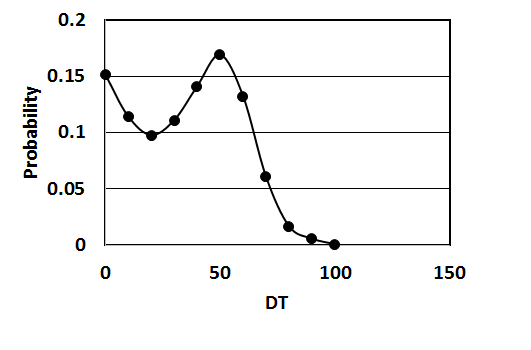


Figure S2. Probability distribution *P* (*DT*) of the interdrop interval *DT* using the TM-stable model.


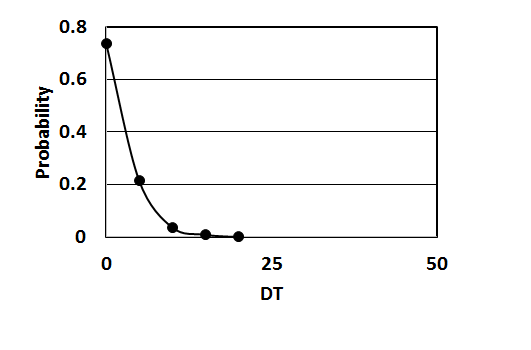


Figure S3. Probability distribution *P* (*DT*) of the interdrop interval *DT* using the TM-unstable model.


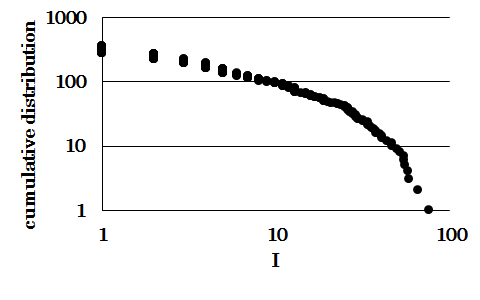


Figure S4. The relationship between increment *I* (*n*) and cumulative distribution of *I* (*n*) when entering position for each agent is changed from {(500, 500)} to {(500, 500), (500, 499), (500,498)}.
